# Comparison of measures of diet quality using 24-hour recall data of First Nations adults living on reserves in Canada

**DOI:** 10.17269/s41997-021-00489-5

**Published:** 2021-06-28

**Authors:** Malek Batal, Hing Man Chan, Amy Ing, Karen Fediuk, Peter Berti, Tonio Sadik, Louise Johnson-Down

**Affiliations:** 1 Département de nutrition, Faculté de Médecine, Pavillon Liliane de Stewart, Université de Montréal, CP 6128 succ. Centre-Ville, Montréal, QC H3T 1A8 Canada; 2 Centre de recherche en santé publique de l’Université de Montréal et du CIUSS du Centre-sud-de-l’Île-de-Montréal (CReSP), 7101 Avenue du Parc, Montréal, QC H3N 1X7 Canada; 3 Department of Biology, University of Ottawa, 30 Marie Curie, Ottawa, ON K1N 6N5 Canada; 4 First Nations Food, Nutrition and Environment Study, University of Ottawa, 30 Marie Curie, Ottawa, ON K1N 6N5 Canada; 5 HealthBridge Foundation of Canada, 1 Nicholas Street, Suite 1004, Ottawa, ON KIN 7B7 Canada; 6 Assembly of First Nations, 55 Metcalfe Street, Suite 1600, Ottawa, ON K1P 6L5 Canada

**Keywords:** Indigenous, First Nations, Diet quality, Traditional food, Healthy Eating Index, NOVA, Autochtone, Première Nations, aliments traditionnels, Indice d’alimentation saine, NOVA

## Abstract

**Objective:**

Assess the diet quality of First Nations adults in Canada using percentage energy from traditional foods (TF) and ultra-processed products (UPP), food portions from the 2007 *Eating Well with Canada’s Food Guide - First Nations, Inuit and Métis* (EWCFG-FNIM) and a Healthy Eating Index (HEI).

**Methods:**

Data collection for this participatory research occurred in 92 First Nations reserves across Canada from 2008 to 2016. Percent daily energy intakes were estimated from 24-hour recalls for TF and NOVA food categories. Portions of food groups from the 2007 EWCFG-FNIM were compared to recommendations. A Canadian-adapted HEI was calculated for each participant.

**Results:**

The percent energy from TF was 3% for all participants and 18% for consumers. Meat and alternatives were above the EWCFG-FNIM recommendations and all other food groups were below these. HEI was “low” with only older individuals attaining “average” scores. HEI was above “average” in 4 regions. UPP represented 55% of energy, the largest proportion from a NOVA category.

**Conclusion:**

The diet quality of First Nations adults in Canada is nutritionally poor. The nutrition, food security and health of First Nations would be improved by better access to TF and healthy store-bought food. However, poor diet is only one aspect of the difficulties facing First Nations in Canada. Researchers and policy makers must strive to better understand the multiple challenges facing First Nations Peoples in order to foster empowerment and self-determination to develop First Nations living conditions and lifestyles that are more culturally sound and more conducive to health.

## Résumé

**Objectifs:**

Évaluer la qualité de l’alimentation des adultes des Premières Nations du Canada en calculant l’énergie provenant des aliments traditionnels (AT) et des produits ultra-transformés, en comparant les portions consommées avec celles recommandées dans *Bien manger avec le Guide alimentaire canadien Premières Nations, Inuit et Métis (BMGAC-PNIM)* de 2007 et en mesurant l’indice d’alimentation saine.

**Méthode:**

La collecte de données pour cette recherche participative a eu lieu dans 92 réserves des Premières Nations au Canada entre les années 2008 et 2016. À partir des rappels de 24 heures, la proportion d’énergie provenant des AT pour les individus les ayant consommés ou non a été comparée, les portions des catégories d’aliments proposées par le BMGAC-PNIM ont été comparées aux recommandations, l’indice d’alimentation saine adapté pour le Canada a été calculé pour chaque participant et la proportion d’énergie des produits ultra-transformés a été établie.

**Résultats:**

La proportion d’énergie provenant des AT chez les consommateurs et non-consommateurs était de 3 % et celle pour les consommateurs d’AT était de 18 %. Seuls les viandes et substituts dépassaient les recommandations du BMGAC-PNIM tandis que la consommation de toutes les autres catégories était inférieure aux recommandations. L’indice d’alimentation saine était “bas” avec seulement les individus plus âgés atteignant un score “moyen”. L’indice d’alimentation saine était supérieur à “moyen” dans 4 régions. Les aliments ultra-transformés représentaient 55 % de l’énergie, la plus grande proportion de toutes les catégories NOVA.

**Conclusion:**

La qualité de l’alimentation des Premières Nations n’est pas optimale. Leur alimentation pourrait être améliorée avec un meilleur accès aux AT et aux aliments sains provenant du marché. Une mauvaise alimentation n’est toutefois pas la seule difficulté à laquelle font face les Premières Nations du Canada. Les Peuples des Premières Nations doivent être impliquées avec les chercheurs et les responsables politiques pour mieux comprendre les défis multiples auxquels ils font face et instaurer des conditions de vie qui soient culturellement sécuritaires et plus propices à la santé.

## Introduction

Indigenous Peoples everywhere have been undergoing a nutrition transition as an outcome of global population growth and changes to food networks and supplies (Cunningham 2010). Indigenous Peoples in Canada have been experiencing significantly decreased access to and reliance on traditional foods (TF) obtained through hunting, fishing, gathering and agriculture (Batal et al. 2018b; Kuhnlein 2015; Morrison 2020). This change is largely rooted in the ongoing impacts from colonial assimilation policies which have led to the environmental dispossession of land from First Nations territories and a consequential inability to maintain highly localized sustainable food systems (Adelson 2005; Egeland and Harrison 2013; Morrison 2020; Reading 2018; Turner et al. 2013). The forced separation of Indigenous children from their families and their placement in the residential school system until adulthood in an attempt to “civilize and Christianize Aboriginal children” (Truth and Reconciliation Commission of Canada 2012) further exacerbated the problem as it severed them from their culture, including their food culture (Adelson 2005; Reading 2018).

All of these factors contribute to a high prevalence of poor health and high rates of food insecurity among First Nations households in Canada (Batal et al. 2021a; Kolahdooz et al. 2017; McNally and Martin 2017; Willows et al. 2009). The current socio-economic climate continues to favour large-scale industrialized agriculture, forestry and mining activities that impact TF systems through reductions in available habitat, species diversity and population numbers, along with contamination of TF (Chan et al. 2006; Ford 2012; Kuhnlein 2015; Turner et al. 2013).

TF yield many health benefits: the gathering of TF requires physical activity and they play a significant role in the culture of First Nations Peoples (Vallianatos and Willows 2016). TF also contribute to higher intakes of many nutrients in the diet of First Nations individuals when they are present (Batal et al. 2005; Johnson-Down and Egeland 2013); however, they have been largely replaced by nutrient-poor market foods (Batal et al. 2018b). Estimating the amount of TF and its contribution to the nutrients in the diet of First Nations Peoples provides a way to look at the extent of the nutrition transition and evaluate its impact on potential nutrient deficiencies and susceptibility to infectious and chronic disease (Batal et al. 2021c; Kuhnlein and Receveur 1996). The transition away from TF has contributed to the health challenges facing Indigenous Peoples in Canada, including a rise in obesity and nutrition-related chronic diseases such as type 2 diabetes that has reached epidemic proportions in Indigenous Peoples throughout the world and especially First Nations Peoples in Canada (Batal and Decelles 2019; Kolahdooz et al. 2015; Kolahdooz et al. 2017; McNally and Martin 2017; Vallianatos and Willows 2016).

In recent years, researchers have expanded from investigating single nutrient associations with disease to looking at food-based dietary guidelines (FBDG) and dietary scores that encompass a combination of these guidelines and important nutrients (Arvaniti and Panagiotakos 2008). In Canada, the 2007 *Eating Well with Canada’s Food Guide - First Nations, Inuit and Métis* (EWCFG-FNIM) is a FBDG that was established giving portion size goals for each of four food groups (vegetables and fruit, grain products, milk and alternatives, and meat and alternatives) (Dubois et al. 2000; Health Canada 2007). A recent version of this FBDG in Canada with updated food groupings no longer provides portion sizes but has not been adapted for Indigenous populations (Government of Canada 2019).

As the nutrition burden shifts away from micronutrient deficiencies to excessive energy intakes, interest in using indices of diet quality that include adequacy, variety, balance and moderation has intensified (Arvaniti and Panagiotakos 2008; Garriguet 2009). Dubois et al. (2000) compared three popular indices of diet quality with data from the Quebec Nutrition Survey conducted in 1990—the Diet Quality Index, the Healthy Eating Index (HEI), and the healthy diet indicator—and concluded that the HEI was the most suitable as it uses both the nutrient content and the contribution of food groups to the diet, reflecting existing nutrient recommendations and dietary guidelines. They also found that of the three indicators they compared, the HEI was the score that correlated most with the mean adequacy ratio for each nutrient and with people’s perceptions of their diets (Dubois et al. 2000). Last, the HEI is a continuous measure that makes it easier to interpret (Dubois et al. 2000; Garriguet 2006; Woodruff and Hanning 2010).

NOVA (a name, not an acronym) classifies foods based on the degree of processing, with the most processed identified as ultra-processed products (UPP) (Batal et al. 2018b; Monteiro et al. 2016; Moubarac et al. 2017), and it has been recognized by the Food and Agriculture Organization (Food and Agriculture Organization 2015). UPP are often lower in cost, have a longer shelf life and often provide more energy, thereby making them attractive and more efficient for those with limited incomes and those living in remote locations (Monteiro et al. 2018; Moubarac et al. 2017; Willows et al. 2019). The World Health Organization has suggested that the proportion of UPP in the diet can be used as an indicator of diet quality, and associations between UPP and weight gain and obesity have been observed in Latin America (Pan American Health Organization 2015).

We propose to compare various methods used to assess diet quality using data from the First Nations Food, Nutrition and Environment Study (FNFNES). We estimate the proportion of energy from TF, compare food intakes to the 2007 EWCFG-FNIM (Health Canada 2007), assess HEI (Garriguet 2009), and establish the contribution of UPP to energy (Batal et al. 2018b).

## Methods

### Sampling and community participation

FNFNES is a participatory study of on-reserve First Nations adults living in Canada south of the 60^th^ parallel; recruitment, sampling and participation have been described elsewhere (Batal et al. 2018a; Batal et al. 2018b; Chan et al. 2021). Communities in 7 regions were sampled to be representative of all First Nations adults in each of the Assembly of First Nations regions below the 60^th^ parallel. A second-level randomization took place in selecting households in each community and a third-level randomization was conducted within the household to select the adult respondent (Chan et al. 2021). First Nations principles of Ownership, Control, Access and Possession (OCAP®) were followed (Chan et al. 2021; Schnarch 2004). Informed consent was obtained from all individuals (Chan et al. 2021).

Before research tool development, methodology workshops were conducted in each region with representatives from participating communities and adjustments were made to the methodology in accordance with communities’ needs and specificities. For example, information was sought from workshop participants on the identification of TF in each region and TF lists were updated accordingly. Upon completion of data collection and analysis, knowledge transfer workshops were held in First Nations communities, and leadership and members were presented with their community’s results; these were discussed at length and their representativeness evaluated by community members. Feedback was incorporated before any results were subsequently released to the community in a detailed report with lay summaries and visual aids (pamphlets and short publications). When possible, return of the results accompanied a TF feast organized by the community researchers. Raw data were also returned to each Nation’s leadership and members, and data analysis training was provided to community representatives. No community-specific data were shared beyond the community, and all regional and Canada-wide reports and scientific publications were anonymized and established using aggregate data.

### Data collection

Trained First Nations researchers, supervised by a registered dietitian, conducted interviews with on-reserve First Nations participants in the fall months of 2008 to 2016. Diet was measured using a 24-hour recall that excluded alcohol intake (Batal et al. 2021c) and used 3-stage multiple-pass method: the respondent was asked to provide a quick list of the foods consumed, then a detailed description of the foods and the portion size, followed by a final review (Raper et al. 2004). Three-dimensional food models were used to help respondents estimate portion sizes (Santé Québec, Montréal, QC).

### Data entry and analysis

Epi Info 3.5.4 (Centers for Disease Control and Prevention, Atlanta, Georgia, USA, 1988) was used to enter interview data. CANDAT (Godin, London, ON), a nutrient analysis software that uses the 2010 Canadian Nutrient File (CNF) (Health Canada 2010) and additional databases developed by users, was utilized to enter 24-hour recalls by research nutritionists at the Université de Montréal. A subsample of 10% of the 24-hour recalls was verified for accurate data entry (Batal et al. 2021c).

Twenty-four-hour recalls were obtained from 6487 individuals. The data of 286 individuals were excluded (245 pregnant and/or lactating women, 27 participants with missing age and age group values, and 14 participants with zero kcal intake), leaving recalls from 6201 individuals. The percent energy from TF was estimated from the 24-hour recalls. TF were identified for each of the various regions by consultation with First Nations informants and consisted of foods that were hunted, fished, gathered and cultivated. Because agricultural practices differ across regions, some cultivated foods such as berries, potatoes, beans and squash were classified as TF in regions where this was the case.

Diet quality of First Nations adults was assessed in 3 different ways. First, using recommendations from the 2007 EWCFG-FNIM (Health Canada 2007), the quantity of each food in the 24-hour recalls was compared to a standard portion size from one of the four food groups: grain products, milk and alternatives, meat and alternatives, and vegetables and fruit. Mixed foods that contained foods from two or more food groups were disassembled to compare them to standard portion sizes for the food groups they contained. The proportion of energy from food groups was also calculated.

Second, diet quality was assessed using a Canadian HEI, a tool adapted from the American HEI to gauge how closely the foods eaten by Canadians follow recommendations outlined in the 2007 EWCFG-FNIM, and other food and nutrient-related components were estimated for each individual (Garriguet 2009; Health Canada 2007). The HEI has been identified as a suitable score to investigate the diet quality and it has been tested for both content and construct validity (Guenther et al. 2008).

The HEI score (maximum total score of 100) is comprised of 8 adequacy components and 3 moderation components (Table 1) (Garriguet 2009; Steinhouse 2017). Proportional points were assigned for criteria falling between the minimum and maximum scores (Table 1). The SIDE (Software for Intake Distribution Estimation) SAS subroutine (Iowa State University, Ames, Iowa, 2001) was used to estimate the population distribution of HEI. Based on the HEI total scores, diet quality was categorized into the following intervals: “low” (less than 50 points), “average” (50–80 points) and “high” (more than 80 points) (Garriguet 2009).

**Table 1:** Components and scoring of a Canadian Healthy Index Score^a^

Components	Criteria for maximum score by gender and age^b^				Criteria for minimum score	Maximum score (points)
	Women, 19–50 y	Men, 19–50 y	Women, >50 y	Men, >50 y		
Adequacy						
Total fruits and vegetables (servings)	7	8	7	7	0	10
Whole fruits (servings)	1.5	2	1.5	1.5	0	5
Dark green and orange vegetables (servings)	1.5	2	1.5	1.5	0	5
Total grain products (servings)	6	8	6	7	0	5
Whole grains (servings)	3	4	3	3.5	0	5
Milk and alternatives (servings)	2	3	2	3	0	10
Meat and alternatives (servings)	2	3	2	3	0	10
Unsaturated fats^c^ (g)	30	45	30	45	0	10
Moderation						
Saturated fat (percent of energy)	7	7	7	7	≥15	10
Sodium (mg)	≤1500	≤1500	≤1500	≤1500	≥4600	10
Other foods^d^ (percent of energy)	≤5	≤5	≤5	≤5	≥40	20

^a^Based on Garriguet (2009)

^b^Based on *Eating Well with Canada’s Food Guide - First Nations, Inuit and Métis* (Health Canada 2007)

^c^Non-hydrogenated oils

^d^Foods not included in *Eating Well with Canada’s Food Guide - First Nations, Inuit and Métis* (Health Canada 2007)

Last, NOVA categories were also used to estimate diet quality. NOVA assigns foods to 4 categories based on the degree of processing: fresh or minimally processed, processed culinary ingredients, processed foods, and UPP (Batal et al. 2018b). Each food in the 24-hour recalls was classified according to NOVA criteria and the percent of energy was calculated for UPP vs. non-UPP.

### Statistical analysis

All data analyses used SAS/STAT version 9.4 (SAS, Cary, NC, USA, 2013). All analyses were weighted for the contribution of the community, household and individual, non-response and population changes over the course of the study from 2008 to 2017.

## Results

**Fig. 1:**
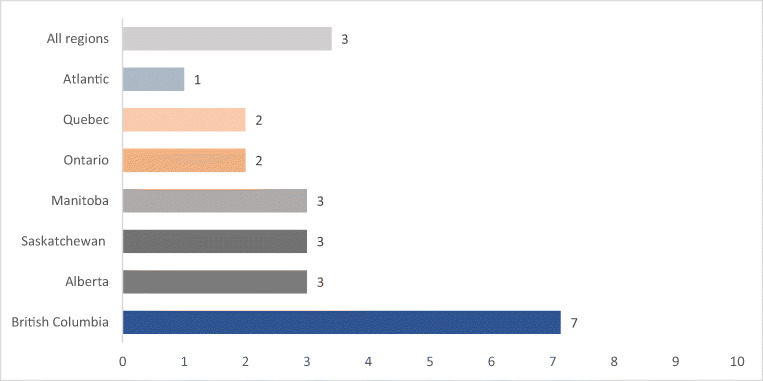
Percent energy from traditional food of 6201 adults 19 years and older from 92 on-reserve First Nations communities in Canada, 2008–2018

Eighteen percent of the 24-hour recalls contained at least one TF (mainly from fish/seafood, plants, land mammals and wild birds). Across Canada, the percent energy from TF was just above 3% for all participants and 18% for actual TF consumers (Fig. 1). In high consumers (95th percentile), more than half (53%) of total energy was derived from TF. Energy from TF was greater in British Columbia (7%) than in the more Eastern regions of Ontario (2%), Quebec (2%) and Atlantic (1%) (Fig. 1). Energy from TF in the Atlantic region was lower than that in Saskatchewan, Alberta and British Columbia (Fig. 1).

When compared with the 2007 EWCFG-FNIM, First Nations participants in all the regions did not meet the recommendations for any of the four food groups (Health Canada 2007). Vegetables and fruit and milk and alternatives intakes in both women and men were less than half of recommended servings in all regions (Table 2). Grain products approached the recommended servings but were not met in any region (Table 2). Meat and alternatives exceeded the recommended servings by greater than 30% and would have included TF (Table 2). Numbers of servings of vegetables and fruit were greater in women but not men in British Columbia as compared with those in Saskatchewan and Atlantic regions (Table 2). Men in Ontario reported more servings of grain products as compared with men in British Columbia (Table 2).

**Table 2:** Mean number of *Eating Well with Canada’s Food Guide - First Nations, Inuit and Métis* servings compared with recommendations in 6201 adults 19 years and older from 92 on-reserve First Nations communities in Canada, 2008–2018

			**Mean number of servings per day (95% CI)**							
**Canada’s Food Guide Recommended number of servings/day**			**All regions (*n*=4010)**	**British Columbia (*n*=652)**	**Alberta (*n*=349)**	**Saskatchewan (*n*=673)**	**Manitoba (*n*=451)**	**Ontario (*n*=855)**	**Quebec (*n*=392)**	**Atlantic (*n*=638)**
Women	7–8	Vegetables and fruit	2.8 (2.7, 2.9)	3.2 (3.1, 3.4)	2.7 (2.3, 3.2)	2.5 (2.3, 2.7)	2.6 (2.1, 3.1)	2.7 (2.3, 3.1)	2.9 (2.6, 3.1)	2.6 (2.5, 2.8)
	6–7	Grain products	4.9 (4.6, 5.2)	4.3 (3.4, 5.2)	5.2 (4.4, 6.0)	5.1 (4.4, 5.8)	5.0 (4.2, 5.7)	4.7 (4.3, 5.1)	5.5 (4.9, 6.1)	4.4 (4.2, 4.7)
	2–3	Milk and alternatives	0.8 (0.8, 0.9)	0.8 (0.7, 1.0)	0.8 (0.5, 1.1)	0.6 (0.5, 0.8)	0.8 (0.6, 1.0)	1.0 (0.8, 1.2)	0.8 (0.8, 0.9)	0.9 (0.8, 1.0)
	2	Meat and alternatives	3.0 (2.8, 3.2)	3.1 (2.8, 3.4)	3.2 (2.7, 3.7)	2.8 (2.5, 3.1)	3.0 (2.5, 3.5)	3.1 (2.6, 3.5)	3.0 (2.8, 3.2)	2.3 (2.1, 2.4)
**Canada’s Food Guide Recommended number of servings/day**			**All regions (*n*=2191)**	**British Columbia (*n*=394)**	**Alberta (*n*=218)**	**Saskatchewan (*n*=317)**	**Manitoba (*n*=229)**	**Ontario (*n*=531)**	**Quebec (*n*=153)**	**Atlantic (*n*=349)**
Men	7–10	Vegetables and fruit	3.0 (2.8, 3.3)	3.4 (2.5, 4.3)	2.8 (2.4, 3.2)	3.0 (2.5, 3.5)	2.9 (2.4, 3.3)	3.0 (2.7, 3.3)	3.1 (2.2, 4.1)	2.9 (2.6, 3.2)
	7–8	Grain products	5.9 (5.4, 6.3)	4.8 (4.0, 5.7)	5.6 (4.6, 6.7)	7.0 (5.3, 8.7)	5.9 (5.5, 6.2)	6.3 (5.9, 6.8)	6.3 (2.7, 9.9)	5.5 (4.9, 6.1)
	2–3	Milk and alternatives	1.0 (0.9, 1.1)	0.8 (0.5, 1.1)	0.9 (0.8, 1.1)	1.0 (0.8, 1.2)	0.9 (0.6, 1.2)	1.1 (1.0, 1.3)	0.9 (0.7, 1.0)	1.1 (0.9, 1.3)
	3	Meat and alternatives	4.0 (3.7, 4.3)	4.0 (3.4, 4.6)	4.2 (3.7, 7.8)	4.3 (3.7, 4.9)	3.9 (3.1, 4.8)	4.1 (3.5, 4.6)	3.7 (2.5, 4.8)	3.1 (2.9, 3.3)

*CI*, confidence interval

The HEI for all regions was “low” for both men and women, but in some regions an “average” HEI was attained. HEI was “low” in 54% of participants, 48% had “average” and less than 1% had “high” HEI. Women in British Columbia, Ontario and Quebec and men in Ontario had “average” scores (Table 3). The mean HEI for both men and women aged 19–50 was “low” (Table 3) while the score for men and women 51 years and older was “average”. Less than 1% of First Nations participants had an HEI greater than 80 points.

**Table 3:** Mean Healthy Eating Index^a^ in 6201 adults 19 years and older from 92 on-reserve First Nations communities in Canada, 2008–2018

	**Healthy Eating Index (95% CI)**							
	**All regions (*n*=4010)**	**British Columbia (*n*=652)**	**Alberta (*n*=349)**	**Saskatchewan (*n*=673)**	**Manitoba (*n*=451)**	**Ontario (*n*=855)**	**Quebec (*n*=392)**	**Atlantic (*n*=638)**
Women	49.7 (49.0, 50.5)	50.8 (49.1, 52.4)	49.7 (47.6, 51.7)	48.8 (47.8, 49.8)	47.4 (45.4, 49.4)	51.0 (49.3, 52.7)	51.3 (49.0, 53.5)	48.4 (47.2, 49.5)
	**All regions (*n*=2191)**	**British Columbia (*n*=394)**	**Alberta (*n*=218)**	**Saskatchewan (*n*=317)**	**Manitoba (*n*=229)**	**Ontario (*n*=531)**	**Quebec (*n*=153)**	**Atlantic (*n*=349)**
Men	47.8 (46.4, 49.2)	48.4 (44.9, 51.9)	45.6 (44.2, 47.0)	49.6 (48.2, 50.9)	46.0 (41.6, 50.4)	50.8 (49.4, 52.1)	45.2 (36.7, 53.7)	47.2 (45.9, 48.5)

*CI*, confidence interval

^a^Based on Garriguet (2009)

Energy from UPP averaged 55% in these First Nations adults. Mean energy from NOVA categories followed a similar pattern across genders and regions with UPP representing the largest proportion and processed foods the smallest proportion (Table 4). Men reported more energy from UPP than women, but the proportion of energy was similar. The mean energy from fresh or minimally processed foods was lower in women in the Atlantic region as compared with women in Quebec, Ontario, Saskatchewan, Alberta and British Columbia, and was also lower in men in the Atlantic region as compared with men in Ontario and Saskatchewan. In women but not men, energy from UPP was higher in Manitoba, Ontario, Quebec and Atlantic regions than in British Columbia.

**Table 4:** Mean energy from non-ultra-processed products (non-UPP) and ultra-processed products (UPP) of 6201 adults 19 years and older from 92 on-reserve First Nations communities in Canada, 2008–2018

		**Energy (kcal) (95% CI)**							
	**NOVA category**	**All regions (*n*=4010)**	**British Columbia (*n*=652)**	**Alberta (*n*=349)**	**Saskatchewan (*n*=673)**	**Manitoba (*n*=451)**	**Ontario (*n*=855)**	**Quebec (*n*=392)**	**Atlantic (*n*=638)**
Women	Fresh or minimally processed foods	624 (584, 665)	654 (537, 771)	685 (583, 786)	604 (542, 665)	610 (497, 722)	576 (532, 620)	676 (573, 779)	487 (455, 519)
	Processed culinary ingredients	104 (93, 115)	93 (64, 122)	120 (87, 152)	120 (92, 148)	111 (78, 145)	95 (82, 108)	89 (69, 108)	102 (84, 120)
	Processed foods	77 (68, 87)	99 (61, 137)	75 (40, 111)	62 (45, 78)	61 (45,76)	85 (69, 101)	79 (63, 96)	68 (59, 77)
	UPP	992 (950, 1033)	862 (811, 913)	971 (858, 1084)	918 (795, 1041)	1088 (969, 1206)	1058 (954, 1162)	1065 (977, 1152)	1046 (978, 1115)
	**NOVA category**	**All regions (*n*=2191)**	**British Columbia (*n*=394)**	**Alberta (*n*=218)**	**Saskatchewan (*n*=317)**	**Manitoba (*n*=229)**	**Ontario (*n*=531)**	**Quebec (*n*=153)**	**Atlantic (*n*=349)**
Men	Fresh or minimally processed foods	750 (697, 802)	742 (578, 906)	714 (613, 815)	883 (786, 980)	711 (569, 853)	820 (723, 916)	643 (515, 770)	644 (571, 716)
	Processed culinary ingredients	127 (115, 139)	119 (75, 162)	151 (117, 186)	113 (92, 134)	124 (98, 151)	129 (107, 150)	121 (83, 158)	126 (100, 151)
	Processed foods	82 (70, 94)	94 (53, 135)	78 (51, 104)	76 (43, 109)	73 (50, 95)	82 (60, 103)	93 (50, 136)	76 (65, 88)
	UPP	1208 (1125, 1291)	1047 (862, 1231)	1333 (1103, 1563)	1118 (903, 1333)	1199 (936, 1462)	1207 (1060, 1353)	1378 (1283,1472)	1320 (1216, 1424)

*CI*, confidence interval; *UPP*, ultra-processed products

## Discussion

All the methods used in this study to assess diet quality yielded similar results, showing that the diet consumed by First Nations adults living on reserves in Canada can be characterized as nutritionally inadequate to meet the needs of the population. The widely observed high burden of nutrition-related chronic disease in this population is a compelling indicator that diet improvements are needed, and such improvements are only likely if there is a system-wide transformation to improve access to healthy foods (Batal and Decelles 2019; Kolahdooz et al. 2017; McNally and Martin 2017).

 Traditional food has well-documented cultural and health benefits beyond nutrition, including both the mental and physical benefits from harvesting with family and friends (Vallianatos and Willows 2016). TF intake was higher in British Columbia (7%) than in many of the other provinces (1–3%). It would be interesting to explore why this is the case; have First Nations Peoples in British Columbia done a better job at promoting the intake of these important foods? More households also reported TF activities in this region (Batal et al. 2021b). The revitalization of TF systems has the potential to positively influence nutrient intakes and reduce the high food insecurity burden in Indigenous communities (Batal et al. 2021b; Centre for Indigenous Conservation and Development Alternatives 2017; Morrison 2006, 2011). As there is the potential that certain TF can have elevated levels of contaminants that are harmful from either naturally occurring processes or environmental contamination, it is important to be aware of any current evidence and public health directives that may affect their use among more vulnerable populations such as women of childbearing age (Berner et al. 2016; Laird et al. 2013; Majowicz et al. 2016).

We observed low intakes of vegetables and fruit as well as milk and alternatives in this population of First Nations adults (Health Canada 2007), although women in British Columbia reported more servings of vegetables and fruit than women in the other regions. On average, with the exception of British Columbia (3.2 servings in women and 3.4 servings in men) and men in Quebec (3.1 servings), individuals reported 3 or less servings of vegetables and fruit and less than 1.1 servings of milk and alternatives whereas most adult Canadians reported at least 5.2 servings of vegetables and fruit and 1.25 servings of milk and alternatives in the 2015 Canadian Community Health Survey (CCHS) (Garriguet 2019). Only 19% of First Nations adults in our study met the requirements of 5 or more servings of vegetables and fruit as compared with 30% of Canadians in 2016 (Statistics Canada 2019). The cost of foods may be a factor in these results as many First Nations Peoples live in remote areas where food costs are higher (Batal et al. 2021a). Serving sizes for intake of vegetables and fruits and milk and alternatives may not be appropriate for First Nations Peoples as TF could be providing the nutrients expected from these foods.

In the general Canadian adult population aged 19 years and older, the mean HEI score was “average” (Garriguet 2009), while it was less than “average” in this representative sample of First Nations adults living below the 60^th^ parallel in Canada. The better scores in older individuals may reflect a greater intake of TF and underscore the importance of these foods (Batal et al. 2021b). The HEI has been identified as a suitable score to investigate diet quality and the Canadian version developed by Garriguet (2009) mirrors the one developed by the United States Department of Agriculture (Guenther et al. 2008). It is difficult to compare results of the HEI in this study to others as the methodology for calculating the score varies from study to study. One study among the Cree of northern Quebec reports a mean of 36 out of a possible total of 110 (Lavigne-Robichaud et al. 2018). Similarly, other Indigenous Peoples in Canada and throughout the world report poor eating habits (Bauer et al. 2012; Chong et al. 2019; Huet et al. 2012). One weakness of the HEI as it is used in Indigenous Peoples is that it does not distinguish TF sources of protein because it was designed for non-Indigenous populations.

The proportion of energy from UPP in the diet is a proxy measure identifying intake of foods of low nutritional value, high-energy density and high-sugar content (Monteiro et al. 2018). Among First Nations adults in this study, UPP contributed 55% of dietary energy, compared to 47% among Canadian adults in the 2015 CCHS (Nardocci et al. 2019b). In Canada, individuals with higher intakes of UPP are more likely to be obese and to have diabetes and high blood pressure (Nardocci et al. 2019a; Nardocci et al. 2019b). In one First Nation in Quebec, UPP intake is associated with metabolic syndrome (Lavigne-Robichaud et al. 2018).

It has been proposed that the degree of processing may also indicate the quality of the diet (Monteiro et al. 2016; Moubarac et al. 2017) while others suggest that this provides no additional evidence beyond those that link nutrients and chronic disease (Gibney et al. 2017). Technological advances in the food industry have led to increased availability of these highly processed products that are high in refined carbohydrates and fat (Popkin et al. 2006). Previous research in a subset of this population has identified that the portion of the diet coming from UPP has a poorer nutritional value (Batal et al. 2018b). As there is an association between high intakes of UPP and negative health outcomes, including obesity and the metabolic syndrome (Batal et al. 2018b; Johnson-Down et al. 2015; Lavigne-Robichaud et al. 2018; Nardocci et al. 2019b), it is a concern that the proportion of these foods was high in this population. It is unclear however whether the associations of UPP and health are due to the foods themselves or the poor nutrient profile of these foods (Ludwig et al. 2019).

Our study was the first cross-Canada study of First Nations adults below the 60^th^ parallel and fulfilled a need for data in a population under-represented in the literature (Chan et al. 2021). The sample size was large with 6201 participants and analyses were weighted to provide representative results of the population studied. The 24-hour recalls followed a previously validated and standardized open-ended method that may help overcome the danger of underestimation in food intake (Food and Agriculture Organization 2015; Raper et al. 2004; Skinner et al. 2013), and recalls have been used in other large nutrition studies of diet in Canada for assessing diet quality (Garriguet 2009; Garriguet 2019; Nardocci et al. 2019b).

The fall data collection may have influenced the TF intake as it is often seasonal, and availability of foods may vary from year to year. Food intake was self-reported and thereby is subject to bias where foods seen as beneficial such as TF or vegetables and fruit may be overreported and foods perceived as unhealthy may be underreported (Skinner et al. 2013; Willett 2012). Because of a high prevalence of overweight/obesity in this population, an underestimation of energy intake might be expected (Willett 2012). Our nutrient analysis software did not allow for disaggregation of mixed foods into their ingredients, making it necessary to approximate these and their contribution to the 2007 EWCFG-FNIM (Health Canada 2007). There is also difficulty in accurately classifying foods to the correct NOVA categories as mixed foods often contain foods in more than one category and the CNF does not always distinguish between homemade and industrial foods (Batal et al. 2018b; Health Canada 2010).

## Conclusion

We have demonstrated that there is a critical need to achieve a better diet quality of First Nations Peoples in Canada to ameliorate health inequities. Increasing the intake of very important traditional foods in a sustainable manner can improve both their food and nutrient intake (Batal et al. 2021c). Better access to TF and healthy store-bought market foods must be established to help improve the health and food security/sovereignty of First Nations Peoples (Batal et al. 2021a; Batal et al. 2021d). This access can be achieved by addressing the more systemic political, regulatory, environmental and economic barriers to improve access to TF and healthy store-bought market foods. Poor diet is only one aspect of the difficulties facing First Nations Peoples in Canada; researchers and policy makers must strive to better understand the multiple challenges facing First Nations Peoples in order to foster empowerment and self-determination to develop living conditions and lifestyles that are more culturally sound and more conducive to health.

## Data Availability

Data are owned by each participating community. The Assembly of First Nations is data custodian and any requests will be addressed to AFN through the corresponding author.

## References

[CR1] Adelson N (2005). The embodiment of inequity: health disparities in aboriginal Canada. Canadian Journal of Public Health.

[CR2] Arvaniti F, Panagiotakos DB (2008). Healthy indexes in public health practice and research: a review. Critical Reviews in Food Science and Nutrition.

[CR3] Batal M, Decelles S (2019). A scoping review of obesity among Indigenous Peoples in Canada. Journal of Obesity.

[CR4] Batal M, Gray-Donald K, Kuhnlein HV, Receveur O (2005). Estimation of traditional food intake in indigenous communities in Denendeh and the Yukon. International Journal of Circumpolar Health.

[CR5] Batal M, Johnson-Down L, Moubarac JC, Ing A, Fediuk K, Sadik T (2018). Sociodemographic associations of the dietary proportion of ultra-processed foods in First Nations peoples in the Canadian provinces of British Columbia, Manitoba, Alberta and Ontario. International Journal of Food Sciences and Nutrition.

[CR6] Batal M, Johnson-Down L, Moubarac JC, Ing A, Fediuk K, Sadik T (2018). Quantifying associations of the dietary share of ultra-processed foods with overall diet quality in First Nations peoples in the Canadian provinces of British Columbia, Alberta, Manitoba and Ontario. Public Health Nutrition.

[CR7] Batal, M., Chan, H. M., Ing, A., Fediuk, K., Berti, P., Mercille, G., et al. (2021a). First Nations households living on-reserve experience food insecurity: prevalence and predictors among ninety-two First Nations communities across Canada. *Canadian Journal of Public Health, 112*(Supplement 1). DOI: 10.17269/s41997-021-00491-x. 10.17269/s41997-021-00491-x PMC8239078 34181224

[CR8] Batal, M., Chan, H. M., Ing, A., Fediuk, K., Berti, P., Mercille, G., et al. (2021b). Importance of the traditional food systems for First Nations adults living on reserves in Canada. *Canadian Journal of Public Health**, 112*(Supplement 1). 10.17269/s41997-020-00353-y. 10.17269/s41997-020-00353-y PMC8239073 34181221

[CR9] Batal, M., Chan, H. M., Ing, A., Fediuk, K., Berti, P., Sadik, T., et al. (2021c). Nutrient adequacy and nutrient sources of adults among ninety-two First Nations communities across Canada. *Canadian Journal of Public Health, 112*(Supplement 1). 10.17269/s41997-021-00490-y. 10.17269/s41997-021-00490-y PMC8239085 34181222

[CR10] Batal, M., Chan, H. M., Johnson-Down, L., Ing, A., Fediuk, K., Berti, P., et al. (2021d). Associations of health status and diabetes among First Nations Peoples living on-reserve in Canada. *Canadian Journal of Public Health**, 112*(Supplement 1). 10.17269/s41997-021-00488-6. 10.17269/s41997-021-00488-6 PMC8239104 34181230

[CR11] Bauer KW, Widome R, Himes JH, Smyth M, Rock BH, Hannan PJ (2012). High food insecurity and its correlates among families living on a rural American Indian reservation. American Journal of Public Health.

[CR12] Berner J, Brubaker M, Revitch B, Kreummel E, Tcheripanoff M, Bell J (2016). Adaptation in Arctic circumpolar communities: food and water security in a changing climate. International Journal of Circumpolar Health.

[CR13] Centre for Indigenous Conservation and Development Alternatives (2017). Livelihoods, food sovereignty and coping with neoliberal growth. http://cicada.world/research/themes/livelihoods-food-sovereignty-and-coping-with-neoliberal-growth/.

[CR14] Chan HM, Fediuk K, Hamilton S, Rostas L, Caughey A, Kuhnlein H (2006). Food security in Nunavut, Canada: barriers and recommendations. International Journal of Circumpolar Health.

[CR15] Chan, H. M., Fediuk, K., Batal, M., Sadik, T., Tikhonov, C., Ing, A., et al. (2021). The First Nations Food, Nutrition and Environment Study (2008–2018)—rationale, design, methods and lessons learned. *Canadian Journal of Public Health**, 112*(Supplement 1). 10.17269/s41997-021-00480-0. 10.17269/s41997-021-00480-0 PMC8239066 34181220

[CR16] Chong, S. P., Appannah, G., & Sulaiman, N. (2019). Predictors of diet quality as measured by Malaysian Healthy Eating Index among Aboriginal Women (Mah Meri) in Malaysia. *Nutrients, 11*(1). 10.3390/nu11010135. 10.3390/nu11010135 PMC6356371 30634596

[CR17] Cunningham C (2010). Health of indigenous peoples. BMJ.

[CR18] Dubois L, Girard M, Bergeron N (2000). The choice of a diet quality indicator to evaluate the nutritional health of populations. Public Health Nutrition.

[CR19] Egeland G, Harrison GG, Kuhnlein HV, Erasmus B, Spigelski D, Burlingame B (2013). Health disparities: promoting Indigenous Peoples’ health through traditional food systems and self-determination. Indigenous Peoples’ food systems & well-being interventions & policies for healthy communities.

[CR20] Food and Agriculture Organization (2015). Guidelines on the collection of information on food processing through food consumption surveys.

[CR21] Ford JD (2012). Indigenous health and climate change. American Journal of Public Health.

[CR22] Garriguet D, Statistics Canada Health Statistics Division (2006). Overview of Canadians’ eating habits 2004.

[CR23] Garriguet D, Statistics Canada (2009). Diet quality in Canada.

[CR24] Garriguet D, Statistics Canada (2019). Changes in beverage consumption in Canada.

[CR25] Gibney MJ, Forde CG, Mullally D, Gibney ER (2017). Ultra-processed foods in human health: a critical appraisal. The American Journal of Clinical Nutrition.

[CR26] Government of Canada. (2019). Canada’s Food Guide. https://food-guide.canada.ca/en/.

[CR27] Guenther PM, Reedy J, Krebs-Smith SM (2008). Development of the Healthy Eating Index-2005. Journal of the American Dietetic Association.

[CR28] Health Canada. (2007). Eating Well with Canada’s Food Guide - First Nations, Inuit and Métis. http://www.hc-sc.gc.ca/fn-an/alt_formats/fnihb-dgspni/pdf/pubs/fnim-pnim/2007_fnim-pnim_food-guide-aliment-eng.pdf.

[CR29] Health Canada. (2010). Canadian Nutrient File. https://www.canada.ca/en/health-canada/services/food-nutrition/healthy-eating/nutrient-data/canadian-nutrient-file-about-us.html.

[CR30] Huet C, Rosol R, Egeland GM (2012). The prevalence of food insecurity is high and the diet quality poor in Inuit communities. The Journal of Nutrition.

[CR31] Johnson-Down L, Egeland GM (2013). How is the nutrition transition affecting the dietary adequacy in Eeyouch (Cree) adults of Northern Quebec Canada?. Applied Physiology, Nutrition, and Metabolism.

[CR32] Johnson-Down L, Labonte ME, Martin ID, Tsuji LJ, Nieboer E, Dewailly E (2015). Quality of diet is associated with insulin resistance in the Cree (Eeyouch) indigenous population of northern Quebec. Nutrition, Metabolism, and Cardiovascular Diseases.

[CR33] Kolahdooz F, Nader F, Yi KJ, Sharma S (2015). Understanding the social determinants of health among Indigenous Canadians: priorities for health promotion policies and actions. Global Health Action.

[CR34] Kolahdooz F, Sadeghirad B, Corriveau A, Sharma S (2017). Prevalence of overweight and obesity among indigenous populations in Canada: a systematic review and meta-analysis. Critical Reviews in Food Science and Nutrition.

[CR35] Kuhnlein HV (2015). Food system sustainability for health and well-being of Indigenous Peoples. Public Health Nutrition.

[CR36] Kuhnlein HV, Receveur O (1996). Dietary change and traditional food systems of indigenous peoples. Annual Review of Nutrition.

[CR37] Laird BD, Goncharov AB, Egeland GM, Chan HM (2013). Dietary advice on Inuit traditional food use needs to balance benefits and risks of mercury, selenium, and n3 fatty acids. The Journal of Nutrition.

[CR38] Lavigne-Robichaud M, Moubarac JC, Lantagne-Lopez S, Johnson-Down L, Batal M, Laouan Sidi EA (2018). Diet quality indices in relation to metabolic syndrome in an Indigenous Cree (Eeyouch) population in northern Quebec, Canada. Public Health Nutrition.

[CR39] Ludwig DS, Astrup A, Bazzano LA, Ebbeling CB, Heymsfield SB, King JC (2019). Ultra-processed food and obesity: the pitfalls of extrapolation from short studies. Cell Metabolism.

[CR40] Majowicz SE, Meyer SB, Kirkpatrick SI, Graham JL, Shaikh A, Elliott SJ (2016). Food, health, and complexity: towards a conceptual understanding to guide collaborative public health action. BMC Public Health.

[CR41] McNally M, Martin D (2017). First Nations, Inuit and Metis health: considerations for Canadian health leaders in the wake of the Truth and Reconciliation Commission of Canada report. Healthcare Management Forum.

[CR42] Monteiro C, Cannon G, Levy R, Moubarac JC, Jaime P, Martins AP (2016). NOVA. The star shines bright. World Nutrition.

[CR43] Monteiro CA, Cannon G, Moubarac JC, Levy RB, Louzada ML, Jaime PC (2018). The UN decade of nutrition, the NOVA food classification and the trouble with ultra-processing. Public Health Nutrition.

[CR44] Morrison D (2006). First Annual Interior of BC Indigenous Food Sovereignty Conference Report.

[CR45] Morrison D, Wittman H, Desmarais AA, Wiebe N (2011). Indigenous food sovereignty – a model for social learning. Food sovereignty in Canada: creating just and sustainable food systems.

[CR46] Morrison D, Settee P, Shukla S (2020). Reflections and realities: expressions of food sovereignty in the Fourth World. Indigenous food systems: concepts, cases and conversations.

[CR47] Moubarac JC, Batal M, Louzada ML, Martinez Steele E, Monteiro CA (2017). Consumption of ultra-processed foods predicts diet quality in Canada. Appetite.

[CR48] Nardocci M, Leclerc BS, Louzada ML, Monteiro CA, Batal M, Moubarac JC (2019). Consumption of ultra-processed foods and obesity in Canada. Canadian Journal of Public Health.

[CR49] Nardocci, M., Polsky, J., & Moubarac, J. C. (2019b). *How ultra-processed foods affect health in Canada*. Montreal: TRANSNUT, Department of Nutrition, Université de Montréal. https://nutrition.umontreal.ca/wp-content/uploads/sites/45/2019/06/27-june-2019-Consumption-of-ultra-processed-foods-and-chronic-diseases-in-Canadian-adults.pdf.

[CR50] Pan American Health Organization (2015). Ultra-processed food and drink products in Latin America.

[CR51] Popkin BM, Armstrong LE, Bray GM, Caballero B, Frei B, Willett WC (2006). A new proposed guidance system for beverage consumption in the United States. The American Journal of Clinical Nutrition.

[CR52] Raper N, Perloff B, Ingwersen L, Steinfeldt L, Anand J (2004). An overview of USDA’s dietary intake data system. Journal of Food Composition and Analysis.

[CR53] Reading CL, Greenwood M, de Leeuw S, Lindsay NM (2018). Structural determinants of Aboriginal Peoples’ health. Determinants of Indigenous Peoples’ Health Beyond the Social.

[CR54] Schnarch, B. (2004). Ownership, control, access, and possession (OCAP) or self-determination applied to research: a critical analysis of contemporary First Nations research and some options for First Nations communities. *Journal of Aboriginal Health, 2004*, 80–95.

[CR55] Skinner K, Hanning RM, Desjardins E, Tsuji LJ (2013). Giving voice to food insecurity in a remote indigenous community in subarctic Ontario, Canada: traditional ways, ways to cope, ways forward. BMC Public Health.

[CR56] Statistics Canada (2019). Fruit and vegetable consumption, 2017.

[CR57] Steinhouse L (2017). The Association between food security and diet quality among First Nations living on-reserve in Canada.

[CR58] Truth and Reconciliation Commission of Canada (2012). They came for the children: Canada, Aboriginal Peoples, and residential schools.

[CR59] Turner, N. J., Plotkin, M., & Kuhnlein, H. V. (2013). Global environmental challenges to the integrity of Indigenous Peoples’ food systems. In B. E. Harriet, V. Kuhnlein, D. Spigelski, & B. Burlingame (Eds.), *Indigenous Peoples’ food systems & well-being: Interventions & policies for healthy communities*. Rome: Food and Agriculture Organization of the United Nations.

[CR60] Vallianatos H, Willows N, Morton J (2016). Tradition and transformation of Eastern James Bay Eeyou (Cree) foodways in pregnancy: implications for health care. Indigenous Peoples: perspectives, cultural roles and health care disparities.

[CR61] Willett W (2012). Nutritional Epidemiology.

[CR62] Willows ND, Veugelers P, Raine K, Kuhle S (2009). Prevalence and sociodemographic risk factors related to household food security in Aboriginal peoples in Canada. Public Health Nutrition.

[CR63] Willows N, Johnson-Down L, Kenny TA, Chan HM, Batal M (2019). Modelling optimal diets for quality and cost: examples from Inuit and First Nations communities in Canada (1). Applied Physiology, Nutrition, and Metabolism.

[CR64] Woodruff SJ, Hanning RM (2010). Development and implications of a revised Canadian Healthy Eating Index (HEIC-2009). Public Health Nutrition.

